# Effects of Pure Cognitive Training on Gait and Balance in Older Adults: Systematic Review and Meta-Analysis

**DOI:** 10.2196/85070

**Published:** 2026-07-29

**Authors:** Xiaohan Li, Yat Leung Chan, Guoshi Liang, Cheuk Hin Lau, Tsz Yau Yuki Lam, Chak Hang Lam, Tsz Wing Chan, Hoi Kei Cheung, Wayne Lap Sun Chan, Chun Liang Hsu, Freddy Man Hin Lam

**Affiliations:** 1 Department of Rehabilitation Sciences Faculty of Health and Social Sciences The Hong Kong Polytechnic University Kowloon China (Hong Kong); 2 Department of Allied Health School of Health Sciences Swinburne University of Technology Victoria Australia

**Keywords:** computerized cognitive training, older adults, aging, physical performance, balance, gait

## Abstract

**Background:**

Physical and cognitive function, both of which decline with aging, are significantly interrelated. Although numerous studies have investigated the effect of physical exercise on cognitive function, relatively few have examined the impact of pure cognitive training on physical performance.

**Objective:**

This review aimed to summarize the effects of pure cognitive training on balance and mobility in older adults.

**Methods:**

Electronic databases (PubMed, Embase, CINAHL, and PsycInfo) were searched in February 2025. Randomized controlled trials that investigated the effect of pure cognitive training on balance and mobility in older adults were included. Pure cognitive training refers to a strictly nonphysical approach involving guided practice on a standardized set of cognitive tasks aimed at optimizing cognitive functioning. For the outcomes, balance performance focused on standing balance tests and comprehensive balance assessment scales. Mobility performance primarily focused on gait speed under a single-task condition (ie, walking only), gait speed under a dual-task condition (ie, conducting cognitive tasks while walking), and the Timed Up and Go test. The Physiotherapy Evidence Database (PEDro) scale was used to evaluate methodological quality. The level of evidence for the available outcomes was rated using the Grading of Recommendations Assessment, Development, and Evaluation (GRADE) system. Meta-analyses and sensitivity analyses of studies with high methodological quality were performed if 3 or more studies were obtained.

**Results:**

Fourteen studies were eventually included. The methodological qualities of 8 (57%) studies were rated as good, while those of 6 (43%) studies were rated as fair. A meta-analysis of studies with high methodological quality showed that pure cognitive training has a significant effect on cognitive-motor dual-task gait speed (standardized mean difference [SMD] 0.39, 95% CI 0.04-0.75; n=376 participants; moderate-quality evidence) but not single-task gait speed (SMD 0.12, 95% CI −0.05 to 0.30; n=506 participants; moderate-quality evidence). No significant improvements were found in functional mobility (SMD 0.29, 95% CI −0.60 to 1.18; n=566 participants; low-quality evidence) or balance (SMD 0.18, 95% CI −0.34 to 0.69; n=139 participants; very low-quality evidence).

**Conclusions:**

Cognitive training does not improve balance or mobility under single-task conditions but does improve walking speed under cognitive-motor dual-task conditions in older adults. A cognitive training protocol targeting executive function, consisting of 40- to 60-minute sessions conducted 2 to 3 times per week over a period of 8 to 10 weeks, has been shown to be effective.

## Introduction

Aging is associated with a decline in functional capacity, which could be attributed to decreased physical and cognitive functions. Existing evidence has demonstrated that physical and cognitive functions are closely interrelated. For instance, faster gait speed is positively correlated with better memory, executive function, and overall cognitive abilities [1]. Greater gait variability is independently linked to poorer cognitive performance among older adults regardless of age [2,3]. Higher cognitive function scores, as assessed by the Mini-Mental State Examination (MMSE), independently predicted better performance in functional mobility, independent of age and other clinical factors [4,5], while mild cognitive impairment (MCI) was found to affect specific gait parameters and functional mobility performance [6].

In terms of causality, substantial research has been conducted to evaluate the effect of physical exercise on cognitive functions. One systematic review found that physical exercise reduces global cognitive decline and improves working memory in individuals with MCI or dementia [7]. Another demonstrated that physical exercise enhances cognitive function across older adults with different levels of cognitive performance, ranging from intact cognition to MCI and dementia [8]. The improvement in cognitive function following physical exercise could be attributed to the increased cerebral perfusion and cardiac output during exercise [8]. Furthermore, physical exercise was found to stimulate the secretion of brain-derived neurotrophic factor (BDNF) in the hippocampi, increase the production of myokines such as irisin and insulin-like growth factor-1 (IGF-1), and reduce the accumulation and aggregation of β-amyloid in the brain [9-12]. These physiological changes provide a foundation for cognitive performance. Collectively, these findings underscore the pivotal role of regular physical activity in preserving cognitive functions in older adults.

On the other hand, research has shown that cognitive processing plays an important role in physical functions, particularly gait and balance. Walking requires motor planning and visuospatial perception, which involve executive function, perceptual-motor function, and visual attention [13]. A progressive decline in frontal cognitive functions, such as that seen in Alzheimer disease, may cause concomitant disorganization of the neural networks that control locomotion, leading to impaired gait performance [13,14]. Similarly, balance is closely related to executive function, mental flexibility, and response inhibition. It has been reported that older adults with balance issues performed worse on tasks requiring mental flexibility and response inhibition [15]. Therefore, there has been an increasing interest in the potential of cognitive training to improve physical function. A recent meta-analysis concluded that sequential programs combining cognitive training and exercise effectively enhance both cognitive and physical functions in healthy older adults. However, this review was unable to isolate the specific impact of cognitive training on physical function [16].

To date, a systematic review published in 2018 [17] evaluated the effect of cognitive training without a physical exercise component on physical function. It revealed that cognitive training can improve gait performance under complex task conditions but not under single-task conditions. On the basis of these findings, investigating the effect of pure cognitive training on mobility and balance, alongside gait, would offer a more comprehensive view of its impact on physical function, given that both domains are closely linked to cognition [1,18]. Furthermore, with new studies published in the past few years [19,20], it is timely to consolidate the latest evidence on the effects of cognitive training on physical function in older adults.

Therefore, this systematic review aims to isolate and evaluate the impacts of single-task cognitive training targeting any cognitive function, including computerized cognitive training (CCT) [21], video games, and cognitive remediation, on mobility and balance outcomes in older adults. The findings will provide an updated understanding of the relationship between cognitive training and physical outcomes.

## Methods

### Data Sources and Searches

A systematic review was conducted to investigate the effects of cognitive training on the mobility and balance of healthy older adults. The PRISMA (Preferred Reporting Items for Systematic Reviews and Meta-Analyses) guideline was followed [22]. Searches were performed in 4 electronic databases (PubMed, Embase, CINAHL, and PsycInfo). The initial search was performed on September 8, 2022, and the final search was done on February 7, 2025. The population, intervention, comparison, outcome, and study design (PICOS) framework was followed for the search strategy. The keywords used in the different databases are shown in Multimedia Appendix 1. The protocol for this systematic review was registered with PROSPERO prior to data analysis (CRD42023401100) [23].

### Study Selection

Search records were screened based on their titles and abstracts to exclude irrelevant articles. The full texts of the remaining articles were reviewed based on the PICOS framework, including the following components: participants (people aged ≥65 years), intervention (pure cognitive training), comparison (usual care or health education), outcome (physical performance), and study design (randomized controlled trial [RCT]). Therefore, this review included studies that (1) recruited older adults aged ≥65 years; (2) provided pure cognitive training as a strictly nonphysical approach involving guided practice on a standardized set of cognitive tasks aimed at optimizing cognitive functioning or slowing brain aging [24,25]; (3) included a comparison group receiving no cognitive training; (4) assessed outcomes related to balance, mobility, or gait under single-task conditions, which refer to the execution of a motor task in isolation, and cognitive-motor dual-task conditions, which involve the simultaneous execution of a primary motor task alongside a concurrent cognitive task; and (5) used a RCT study design. Studies were excluded if they (1) included participants with cognitive impairments (eg, dementia or MCI) or neurological diseases known to impair gait (eg, Parkinson disease and stroke) or (2) applied only cognitive-motor dual-task interventions, preventing the specific effects of cognitive training from being isolated.

### Methodological Quality Assessment and Quality of Evidence

The Physiotherapy Evidence Database (PEDro) scale was used to assess the methodological quality of each selected study [26]. The PEDro scale is a formally validated instrument with established reliability and validity for quantifying the methodological quality of RCTs. For articles that were not available on the PEDro website, they were rated independently by 2 of our team members using the PEDro criteria. A higher PEDro score indicates better methodological quality. The studies were then classified as excellent, good, or fair according to their PEDro score (9-10=“excellent,” 6-8=“good,” and 4-5=“fair”). The quality of evidence for the outcomes was rated using the Grading of Recommendations Assessment, Development and Evaluation (GRADE) approach [27-30]. The downgrade and upgrade criteria of GRADE are listed in Multimedia Appendix 2.

### Data Analysis

The effects of cognitive training on the outcomes of interest were categorized into mobility and balance. Meta-analysis was performed if ≥3 studies of similar outcomes were obtained. Random-effects models were used for all meta-analyses to address heterogeneity across studies. The standardized mean difference (SMD) and 95% CI were computed and presented in forest plots (Review Manager version 5.4.1; Cochrane Collaboration). A *P* value of ≤.05 was considered statistically significant. The *I*^2^ index was used to test the heterogeneity of the included studies in each meta-analysis. A separate meta-analysis including studies with high methodological quality (PEDro score ≥6) was performed to avoid contamination of the results by low-quality studies. For meta-analyses with moderate to high heterogeneity (*I*^2^>50%), subgroup analyses were performed if all subgroups could include at least 2 studies [31]. Publication bias was statistically assessed using the Egger regression asymmetry test to detect potential asymmetries in the evidence base if there were >10 studies included in the meta-analysis [32]. A *P* value <.10 on the Egger regression asymmetry test indicated the presence of publication bias [33]. The Egger regression method is a statistical tool used in meta-analyses to detect publication bias by assessing whether smaller studies with less significant findings are underrepresented in the published literature. When publication bias was detected, a trim and fill analysis was conducted to impute the findings of potentially unpublished studies and assess the robustness of the results. Narrative analyses were performed for outcomes for which meta-analyses could not be performed.

## Results

**Figure 1:**
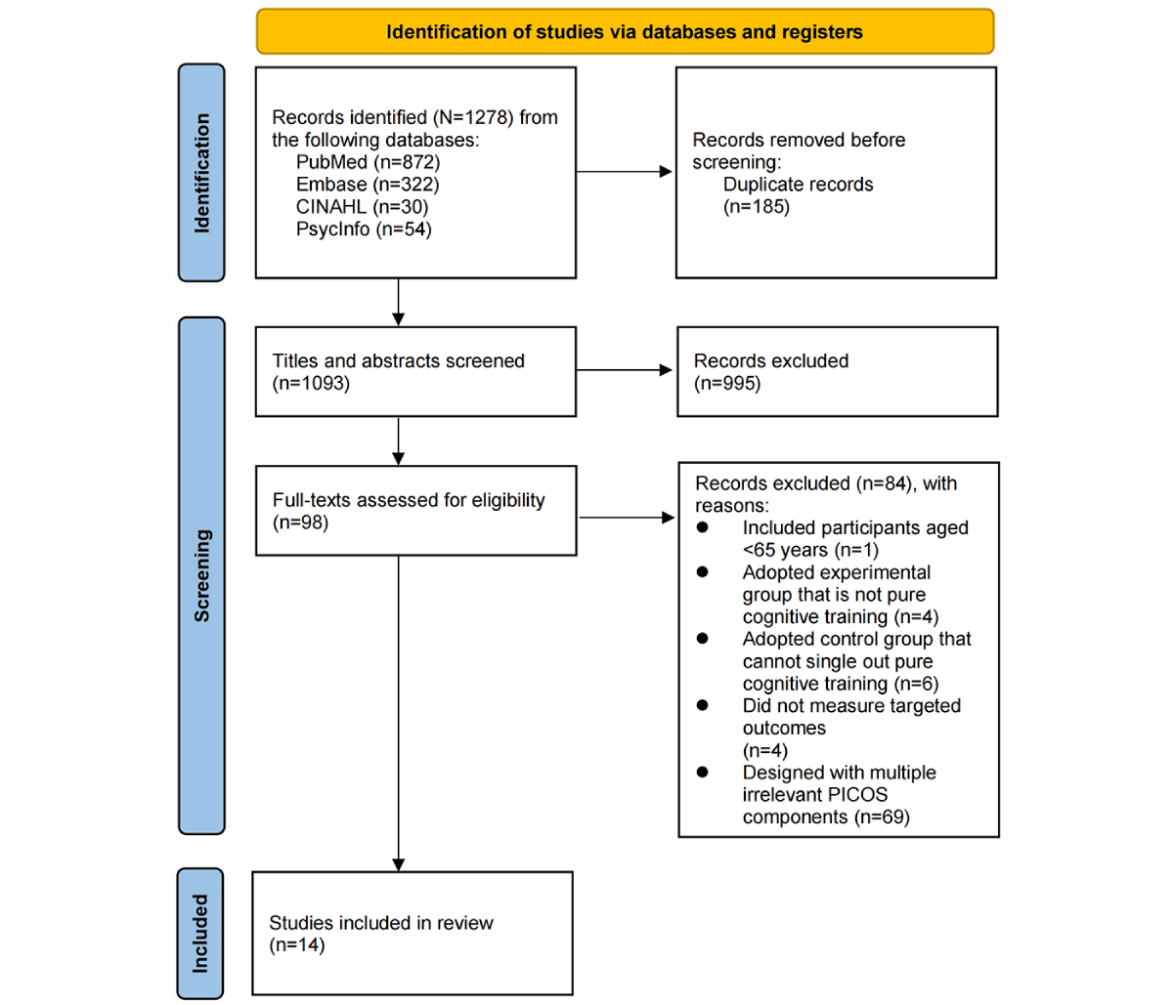
PRISMA (Preferred Reporting Items for Systematic reviews and Meta-Analyses) flow diagram of the study selection process. PICOS: population, intervention, comparison, outcome, and study design; RCT: randomized controlled trial.

Our search yielded a total of 1278 records. Ultimately, 14 (1%) trials [19,20,34-45] with a total of 898 participants met the eligibility criteria and were included in this systematic review (Figure 1).

### Study Characteristics

The characteristics of the included studies are summarized in Table 1. The sample size ranged from 20 to 238, with the mean age of participants ranging from 66.2 (SD 7.1) to 85.7 (SD 5.4) years. Most studies recruited community-dwelling individuals. One study included nursing home residents [42]. One study included only male participants [35]. The details of each study are listed in Multimedia Appendices 3 and 4 [1-14].

**Table 1:** Characteristics of the included studies (N=14).

Characteristics			Studies, n (%)		References
Total sample size					
	<50	8 (57)		[34-38,41-43]	
	50-99	4 (29)		[39,40,44,45]	
	≥100	2 (14)		[19,20]	
Mean age (years)					
	60-64	0 (0)		N/A^a^	
	65-74	5 (36)		[20,37,39,44,45]	
	≥75	9 (64)		[19,34-36,38,40-43]	
Sex					
	Female participants only	0 (0)		N/A	
	Male participants only	1 (7)		[35]	
	Both sexes	13 (93)		[19,20,34,36-45]	
Methodological quality (Physiotherapy Evidence Database scale)					
	<4 (“poor”)	0 (0)		N/A	
	4-5 (“fair”)	6 (43)		[35-37,41,42,45]	
	6-8 (“good”)	8 (57)		[19,20,34,38-40,43,44]	
Source of participants					
	Community-dwelling older adults	12 (86)		[19,20,34,36-41,43-45]	
	Nursing home residents	1 (7)		[42]	
	Not mentioned	1 (7)		[35]	
Types of cognitive training					
	CCT^b^	10 (71)		[19,20,35-38,40,41,43,44]	
	Non-CCT	4 (29)		[34,39,42,45]	
Targeted outcomes^c^					
	Single-task gait speed	13 (93)		[19,34-36,38-44,46,47]	
	Cognitive-motor dual-task gait speed	7 (50)		[19,38,40-44]	
	Functional mobility	6 (43)		[19,20,34,36,40]	
	Static balance (one-leg stance test)	3 (21)		[20,34,37,45]	
	Overall balance ability (balance scales)	3 (21)		[34,41,46]	

^a^N/A: not applicable.

^b^CCT: computerized cognitive training.

^c^The percentages sum to more than 100% as individual studies may report multiple outcomes.

### Intervention Protocols

A total of 71% (10/14) studies used CCT as the intervention [19,20,35-38,40,41,43,44], while 4 (29%) used noncomputerized training [34,39,42,45]. Studies that adopted CCT used training programs designed to improve executive function, which include Mindfit (CogniFit Inc), CogniFit Program (CogniFit Ltd), and other self-designed programs. Noncomputerized training included therapist-led visual attention training [34], attentional capacity and working memory training [39,42], and board game–based cognitive training [45]. The duration of the training programs ranged from 3 weeks to 12 months. Most interventions were conducted 1 to 3 times per week, with each session lasting between 20 and 120 minutes. One study [19] involved self-directed home-based training, in which participants completed flexible sessions at their own pace, averaging 120 to 150 minutes of training per week.

### Methodological Quality and Quality of Evidence

Among the 14 included studies, 8 (57%) [19,20,34,38-40,43,44] were rated as good (PEDro score 6-8) and 6 (43%) [35-37,41,42,45] were rated as fair (PEDro score 4-5). The PEDro score of each article is outlined in Multimedia Appendix 5 [1-14]. The quality of evidence for outcomes rated using GRADE is listed in Table 2. The quality of evidence for gait speed under single-task and cognitive-motor dual-task conditions was rated as moderate. The quality of evidence for functional mobility and balance was rated as low and very low, respectively.

**Table 2:** Grade of Recommendations Assessment, Development, and Evaluation (GRADE) assessment of the quality of evidence^a^.

Outcomes	Risk of bias^b^	Inconsistency	Indirectness	Imprecision	Publication bias	Large effect	Plausible confounding	Dose response gradient	GRADE quality	Effect size (95% CI)
Gait speed	0	0	0	−1^c^	0	0	0	0	Moderate	0.19 (−0.00 to 0.38)
Gait speed (high-quality studies)	0	0	0	−1^c^	0	0	0	0	Moderate	0.12 (−0.05 to 0.30)
Dual-task gait speed	0	0	0	−1^c^	0	0	0	0	Moderate	0.26 (0.03 to 0.49)
Dual-task gait speed (high-quality studies)	0	0	0	−1^c^	0	0	0	0	Moderate	0.39 (0.04 to 0.75)
Functional mobility	0	–1^d^	0	−1^c^	0	0	0	0	Low	0.29 (−0.60 to 1.18)
Balance scale	−1^e^	0	0	−2^b^	0	0	0	0	Very low	0.18 (−0.34 to 0.69)

^a^0 indicates that no serious limitations were identified in that domain; therefore, no downgrading was applied. Negative values (−1 or −2) indicate downgrading of the quality of evidence due to identified limitations (eg, imprecision or inconsistency).

^b^The *P* value of the Egger regression asymmetry test is less than 0.1.

^c^The number of participants included in the primary meta-analysis was <400, and the 95% CI spanned the appreciable harm or benefit thresholds.

^d^Heterogeneity *I*^2^ larger than 50% in the meta-analysis.

^e^More than 50% of the participants included in the primary analysis from trials with a Physiotherapy Evidence Database score of <6.

### Mobility

Fourteen studies [19,20,34-36,38-44,46,47] investigated the effect of cognitive training on mobility-related outcomes.

#### Single-Task Gait Speed

**Figure 2:**
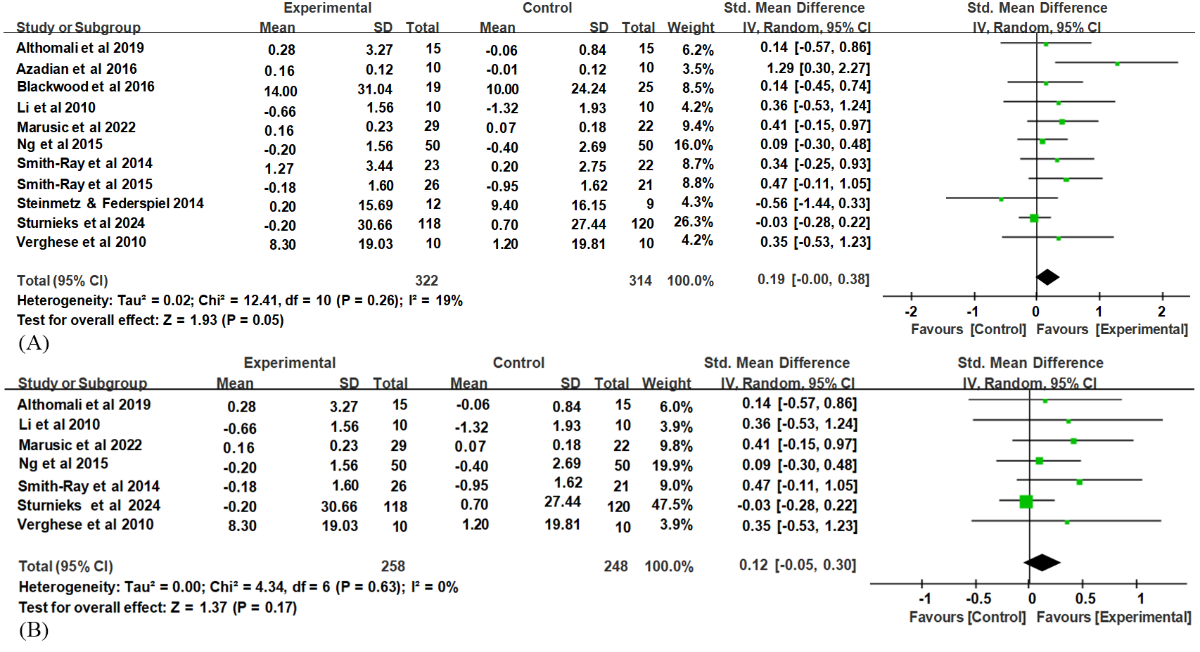
Gait speed. (A) Meta-analysis including all studies that reported gait speed outcomes and (B) meta-analysis including only studies rated as having high methodological quality.

Of the 14 studies, 13 (93%) [19,34-36,38-44,46,47] investigated the effects of cognitive training on gait speed. Meta-analysis showed that cognitive training significantly increased single-task gait speed (SMD 0.19, 95% CI −0.00 to −0.38; *Z*=1.93; *P*=.05; *I*^2^=19%; n=636 participants; moderate-quality evidence; Figure 2A). The Egger regression asymmetry test indicated no evidence of publication bias (*P*=.35). Yet, the result turned nonsignificant in the sensitivity analysis that included only studies with high methodological quality (SMD 0.12, 95% CI −0.05 to −0.30; *Z*=1.37; *P*=.17; *I*^2^=0%; n=506 participants; moderate-quality evidence; Figure 2B) [19,34,38,39,41,43,44].

#### Cognitive-Motor Dual-Task Gait Speed

**Figure 3:**
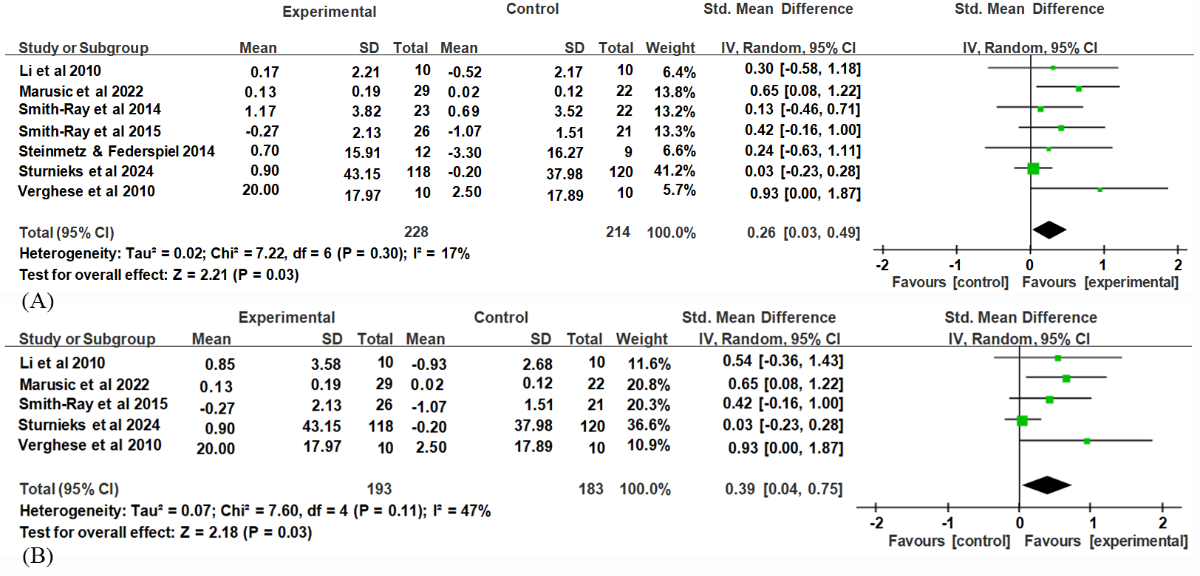
Cognitive-motor dual-task gait speed. (A) Meta-analysis including all studies that reported cognitive-motor dual-task gait speed outcomes and (B) meta-analysis including only studies rated as having high methodological quality.

A total of 50% (7/14) studies [19,38,40-44] investigated the effects of cognitive training on cognitive-motor dual-task gait speed. Meta-analysis showed that cognitive training significantly increased cognitive-motor dual-task gait speed (SMD 0.26, 95% CI 0.03 to −0.49; *Z*=2.21; *P*=.03; *I*^2^=17%; n=442 participants; moderate-quality evidence; Figure 3A). The results remained significant in the sensitivity analysis that included only studies with high methodological quality (PEDro score ≥6; SMD 0.39, 95% CI 0.04 to −0.75; *Z*=2.18; *P*=.03; *I*^2^=47%; n=376 participants; moderate-quality evidence; Figure 3B) [19,38,40,43,44].

#### Functional Mobility

A total of 43% (6/14) studies [19,20,34,36,40,45] investigated functional mobility using the Timed Up and Go (TUG) test as an outcome measure. Meta-analysis showed that cognitive training had no significant effect in improving functional mobility (mean difference [MD] 0.29, 95% CI −0.60 to −1.18; *Z*=0.63; *P*=.53; *I*^2^=77%; n=566 participants; low-quality evidence; Multimedia Appendix 6).

To delineate the contributing factor to the largest heterogeneity, subgroup analyses were conducted based on the methodological quality of the trials, the type of training (CCT vs non-CCT), total training hours of the program, individual training duration, and the baseline mean age of the participants. Nevertheless, the largest *I*^2^ value ranged from 79% to 88% in all subgroup clusters. No clear contributing factor to the high heterogeneity could be identified. The results remained nonsignificant in all subgroup analyses (Multimedia Appendix 7).

Additionally, 7% (1/14) study reported no significant effect on dual-task TUG test (*P*=.53) [34].

#### Other Mobility Parameters

Only 7%(1/14) study [35] reported gait parameters other than gait speed, including stride and step length, stride duration, step, single-support duration, and double-support duration during single-task walking, and gait asymmetry during single-task and dual-task walking. Meta-analysis was not appropriate because of the insufficient number of studies. This study reported statistically significant improvements (*P*<.05) in some gait parameters and a significant decrease in gait asymmetry in the cognitive training group compared with the control group. Detailed results are presented in Multimedia Appendix 4.

### Balance Performance

The effect of cognitive training on balance was evaluated in 5 studies [20,34,37,41,45].

#### Balance Scales

Of the 14 included studies, 2 (14%) used the Berg Balance Scale (BBS) and 1 (7%) used the Mini-Balance Evaluation Systems Test (Mini-BESTest) [34,41,45]. The 2 scales were combined in a meta-analysis. No significant effect was found in the cognitive training group compared with the control group (SMD 0.18, 95% CI −0.34 to −0.69; *Z*=0.67; *P*=.50; *I*²=56%; n=139 participants; very-low–quality evidence; Multimedia Appendix 8). Despite the high heterogeneity, no subgroup analysis could be performed as there were only 3 studies in the meta-analysis. Any grouping would result in at least 1 subgroup containing only 1 study.

#### Static Balance

Only 21% (3/14) studies [20,34,37] reported static balance with contrasting results. The tests were carried out under either eyes-open [34] or eyes-closed [20,37] conditions. Althomali et al [34] reported that cognitive training had no significant effect on the duration of single-leg standing under eyes-open conditions relative to the control group (*P*=.56). Liu et al [20] reported that cognitive training led to a significant increase in the duration of single-leg standing under eyes-closed conditions when compared with the control group (*P*=.005). Hou et al [37] reported only a combined result of the one-leg stance test (OLST) and the TUG test, prohibiting the isolation of the balance outcome for analysis.

## Discussion

### Principal Findings

Our systematic review summarized the effects of cognitive training on mobility and balance in older adults. Overall, our meta-analysis showed that cognitive training significantly enhanced cognitive-motor dual-task gait speed (*P*=.03) and marginally improved single-task gait speed (*P*=.05). However, the results for single-task gait speed turned nonsignificant in the sensitivity analyses that included only studies with high methodological quality. No significant improvement was found in functional mobility or balance outcomes following cognitive training.

### Effectiveness of Cognitive Training on Mobility and Balance Outcomes

#### Overview

Our meta-analysis revealed the effect of cognitive training on balance and mobility performance under different cognitive loads. According to the dual-task paradigm [48], single-task walking, functional mobility tests (ie, TUG test), and balance assessment tests (ie, BBS, Mini-BESTest, and OLST) were considered tasks with a low cognitive load, while cognitive-motor dual-task walking was considered a task with a high cognitive load [49]. Thus, results consolidated in this review suggest that cognitive training could improve only tasks with a high cognitive load and not those with a low cognitive load.

#### Effectiveness of Cognitive Training on Tasks With a High Cognitive Load

One of the possible explanations for this finding is the central capacity sharing model [50]. Cognitive-motor dual-task walking has been described as involving 2 simultaneous tasks (walking and a cognitive task) that interfere with each other and compete for cognitive resources. It has been stated that when attentional resources are limited in capacity, at least one of the tasks may deteriorate in performance. Among the 7 studies reporting cognitive-motor dual-task gait speed outcomes, 4 (57%) [38,40-42] did not assess changes in cognitive performance, 2 (29%) reported no significant change in cognitive function [19,43], and 1 (14%) demonstrated enhanced executive function [44]. Additionally, within the studies [43,44] with a significant improvement in dual-task gait speed, 1 (50%) study showed an improvement in cognitive function [44]. These findings suggest that the observed improvements in gait performance might stem from (1) enhanced cognitive capacity; (2) improved automaticity in performing cognitive tasks [51], allowing more cognitive resources to be allocated to walking, thereby enhancing physical performance without sacrificing cognitive performance; or (3) a mix of both. As we see no deterioration in cognitive performance from the available data, the improved dual-task walking speed would not be the result of a pure reallocation of existing cognitive resources, which would consequently reduce the resources available for concurrent cognitive tasks and potentially diminish cognitive performance [52]. More data are required to confirm our postulation.

#### Effectiveness of Tasks With a Low Cognitive Load

Our results suggest that cognitive training does not improve physical function directly, as seen in the lack of significant changes in outcomes for tasks that require a low cognitive load. Although our overall meta-analysis found a significant improvement in single-task gait speed (*P*=.05), the analysis of studies with high methodological quality did not show a significant improvement. This discrepancy may be attributed to a few factors: the stricter methodological standards of high-quality studies, which likely reduced the potential for overestimated effects, and the lower heterogeneity (*I*^2^=0%) in the analysis of high-quality studies, suggesting more consistent and reliable findings. Meanwhile, studies in the high-quality group generally included larger sample sizes than those of lower quality (506 participants in the high-quality group vs 130 participants in the low-quality group). Notably, 1 large RCT (n=238 participants) reported no significant between-group improvements in single-task gait speed [19]. In contrast, the only study that reported a significant improvement in single-task gait speed had a small sample size, with only 10 participants in each group [35]. Additionally, this study included only male participants and focused on individuals with potential balance impairments, as indicated by a BBS score <52 and/or a self-selected gait speed of ≤1.1 m/s on the 10-meter walk test. These findings suggest that cognitive training may not significantly improve single-task gait speed in relatively healthy older adults.

Nevertheless, our review identified consistent results across outcomes involving low cognitive load, including functional mobility and balance measured using the TUG test, BBS, and Mini-BESTest, with no significant changes observed. We hypothesize that older adults, in general, have sufficient cognitive resources available for less cognitively challenging tasks. Consequently, the central capacity sharing model does not apply [50], which might explain the nonsignificant effect of cognitive training on tasks with a low cognitive load.

#### Conceptual and Mechanistic Interpretation

The pattern of benefit observed in this review can be interpreted within contemporary motor control theory. Healthy adult walking is partly automatic, meaning that steady-state gait can be regulated with limited executive attention, whereas aging and dual-task demands shift gait toward more deliberate, executive control [53,54]. From this perspective, pure cognitive training would not be expected to substantially alter well-practiced single-task walking or simple balance tasks in cognitively intact older adults, as these tasks may already be supported by sufficient automatic sensorimotor control. Its effect is more likely to appear during cognitive-motor dual-task walking, where cognitive resources are invoked, and training-related improvements in attention, inhibition, working memory, or task switching may reduce competition between cognitive and locomotor processes. This also aligns with the near- vs far-transfer framework [55,56]: improvement in dual-task gait speed reflects near transfer, given its shared executive-control demands with cognitive training, while the lack of effects on single-task gait speed, mobility, and balance is consistent with limited far transfer to outcomes with lower cognitive load.

Neurophysiologically, this interpretation is consistent with evidence that dual-task walking engages frontal cortical systems. Functional near-infrared spectroscopy (fNIRS) studies show that walking while talking in older adults is associated with prefrontal cortical activation, supporting the view that attentional and executive-control networks contribute to gait when task demands increase [57]. Systematic neurophysiological evidence also indicates that cognitive-motor interference involves distributed cortical resource allocation rather than a single gait-specific locus [58]. Therefore, the significant improvement in dual-task gait speed found in this review may reflect more efficient frontal compensation or cognitive resource allocation during cognitively demanding walking, although the included trials were not designed to directly test these neural mechanisms. Future trials should combine gait outcomes with neurophysiological measures, such as fNIRS or electroencephalogram (EEG), to determine whether behavioral gains correspond to reduced or more targeted prefrontal recruitment during dual-task gait.

### Effective Training Protocol

As the meta-analysis revealed that pure cognitive training significantly improves only cognitive-motor dual-task gait speed, it is more pertinent to focus on the training protocols that could lead to an improvement in cognitive-motor dual-task gait speed.

Among the studies analyzed for dual-task gait speed with high methodological quality, all used CCT aimed at improving executive function. The training sessions lasted for 40 to 60 minutes, with 2 to 3 sessions per week, for a range of 8 to 10 weeks. After further analysis, we found no difference in training protocols between studies that reported significant improvements [43,44] and those that reported nonsignificant improvements [19,38,40].

### Clinical Significance

This review demonstrated that pure cognitive training significantly improves cognitive-motor dual-task walking speed. Nevertheless, this review provides no direct evidence on whether the small-to-moderate effect size found on dual-task walking speed is clinically important. To contextualize the improvement in dual-task gait speed, a sensitivity analysis, including only studies using the same unit of measurement, was conducted to obtain the MD for comparison with the minimal detectable change (MDC) or minimal clinically important difference (MCID).

Pure cognitive training increased dual-task gait speed by 8.83 cm/s (MD 8.83, 95% CI 0.32-17.34; experimental n=157 participants: control n=152 participants; *I*²=44%; *P*=.04; 3 trials of good methodological quality). This improvement falls within the ranges of MDC (5-10 cm/s) [59,60] and MCID (8-20 cm/s) [61,62] reported for various older populations. Therefore, a firm quantitative conclusion about its clinical significance cannot be drawn. Nevertheless, this improvement might be valuable as cognitive-motor dual-task walking speed is a critical predictor of falls, frailty, disability, and mortality [63].

In particular, the improvement in cognitive-motor dual-task gait speed may be relevant to fall prevention because walking in real-world situations often involves distractions, such as texting while walking [64]. Cognitive-motor dual-task gait speed reflects the ability to maintain walking performance under divided-attention conditions that commonly occur in daily life. A systematic review and meta-analysis reported that slower gait speed under both single- and dual-task conditions can differentiate fallers from nonfallers among older adults, supporting dual-task gait speed as a marker of fall risk [65]. More specifically, in a prospective cohort of 646 community-dwelling older adults, poorer walking-while-talking speed predicted incident falls after adjustment for normal walking velocity and other confounders. Conversely, participants in the fastest 14% of the walking-while-talking speed distribution had a significantly lower risk of incident falls than the remaining 86% of the cohort [66]. Therefore, the gain in cognitive-motor dual-task gait speed may indicate a greater capacity to walk safely while attention is divided between concurrent tasks.

However, pure cognitive training did not appear to improve physical performance directly in older adults. This could be attributed to the low cognitive requirement of simple balance and general mobility tasks among healthier older adults. It should be noted that the cognitive load of the same task could be different in older adults with different abilities. We observed a trend that studies that included older adults with poorer physical function [20,35,40,45], or included more difficult balance tasks (ie, single-leg standing with eyes closed) [20], tended to report significant improvements. Results might differ if more challenging balance tasks were involved or if participants were older adults with known physical or cognitive impairments.

### Limitations

For the 5 trials that included balance outcomes, only 3 types of outcomes (BBS, Mini-BESTest, and OLST) were used. The insufficient diversity in balance outcomes prevents a comprehensive analysis of balance performances, thereby restricting the ability to evaluate the relationship between cognitive training and balance.

This meta-analysis primarily used gait speed and cognitive-motor dual-task gait speed as indicators of gait performance. However, gait performance should not be determined solely by gait velocity. Other gait parameters, such as spatiotemporal measures, can also be an important determining factor of gait performance. Yet, the effects of cognitive training on the spatiotemporal aspects of gait were not examined because of the limited number of studies available. Thus, future investigations are needed to explore the relationship between cognitive training and various spatiotemporal gait characteristics under single-task conditions among healthy older adults.

As this study included only cognitively intact older adults without any diseases affecting gait, the findings may not be generalizable to those with cognitive impairments or gait-related health conditions. Additionally, the cognitive training in most included studies focused primarily on enhancing executive function without targeting other cognitive domains.

Furthermore, for the meta-analysis with high heterogeneity (functional mobility), metaregression analysis was not performed as the meta-analysis included only 6 studies, falling short of the recommended minimum of 10 studies required for a metaregression model [32].

### Conclusions

Cognitive training, aimed at improving executive function and lasting for 40 to 60 minutes per session, 2 to 3 sessions per week, over 8 to 10 weeks, improves cognitive-motor dual-task walking speed in older adults. However, cognitive training could not improve single-task mobility or balance performance in cognitively intact older adults.

## Appendix

Multimedia Appendix 1 Details of search strategies.

Multimedia Appendix 2 Downgrade and upgrade criteria of Grading of Recommendations Assessment, Development, and Evaluation (GRADE).

Multimedia Appendix 3 Summary of participant characteristics and intervention protocols.

Multimedia Appendix 4 Summary of intervention effects.

Multimedia Appendix 5 Physiotherapy Evidence Database (PEDro) score of included articles.

Multimedia Appendix 6 Meta-analysis comparing functional mobility between the intervention group and the control group.

Multimedia Appendix 7 Subgroup meta-analyses for functional mobility to identify the source of heterogeneity.

Multimedia Appendix 8 Meta-analysis comparing balance scales between the intervention group and the control group.

Multimedia Appendix 9 PRISMA 2020 checklist.

## Abbreviations

BBS: Berg Balance Scale
BDNF: brain-derived neurotrophic factor
CCT: computerized cognitive training
EEG: electroencephalogram
fNIRS: functional near-infrared spectroscopy
GRADE: Grading of Recommendations Assessment, Development, and Evaluation
IGF-1: insulin-like growth factor-1
MCI: mild cognitive impairment
MCID: minimal clinically important difference
MD: mean difference
MDC: minimal detectable change
Mini-BESTest: Mini-Balance Evaluation Systems Test
MMSE: Mini-Mental State Examination
OLST: one-leg stance test
PEDro: Physiotherapy Evidence Database
PICOS: population, intervention, comparison, outcome, and study design
PRISMA: Preferred Reporting Items for Systematic Reviews and Meta-Analyses
RCT: randomized controlled trial
SMD: standardized mean difference
TUG: Timed Up and Go

## Data Availability

Data are available from the corresponding author on reasonable request.
