# Supplementary material for: Effects of Pure Cognitive Training on Gait and Balance in Older Adults: Systematic Review and Meta-Analysis
Source: JMIR Aging. 2026 Jul 29;9:e85070. doi: 10.2196/85070 (PMC13469839; doi:10.2196/85070)
Supplement: Supplementary file 1 [file aging_v9i1e85070_app1.docx]

**Table S1.** Details of search strategies

**Databases: CINAHL**

1. MH “cognitive training”
2. TX “cognitive stimulation”
3. TX “cognitive rehabilitation”
4. TX “cognitive remediation”
5. TX “memory training”
6. MH “video games”
7. TX “computer game”
8. TX “attention training”
9. 1 or 2 or 3 or 4 or 5 or 6 or 7 or 8
10. TX “older adults”
11. TX elderly
12. MH aged
13. TX aging
14. TX elders
15. MH “frail elderly”
16. TX geriatric
17. 10 or 11 or 12 or 13 or 14 or 15 or 16
18. TX mobility
19. MH gait
20. TX balance
21. MH “postural balance”
22. TX stability
23. TX fall
24. MH posture
25. 18 or 19 or 20 or 21 or 22 or 23 or 24
26. 9 and 17 and 25

Filters:

Language: English

Publication type: Randomized controlled trial

Age groups: 1. Middle aged: 45-64 years, 2. Aged: 65+ years, 3. Aged, 80 and over

MH: Medical subject headings

TX: Text word

**Databases: PubMed**

1. ((((((("Gait"[Mesh])) OR "Posture·[Mesh]) OR "Postural Balance" [Mesh]) OR (mobility)) OR (balance)) OR (stability)) OR (fall)
2. ((((("Aged"[Mesh]) OR "Frail Elderly"[Mesh]) OR (older adults)) OR (elderly)) OR Caging)) OR (elders)) OR (geriatric)
3. (((((("Video Games"[Mesh]) OR ("cognitive training•[Mesh]) or (cognitive training)) OR (cognitive stimulation)) OR (cognitive Rehabilitation)) OR (memory training)) OR {computer game)) OR (attention training)
4. 1 AND 2 AND 3

Filters:

Language: English

Publication type: Randomized controlled trial

Age groups: Aged: 65+ years, 80 and over: 80+ years

Mesh: Medical Subject Headings

**Databases: EMBASE**

1. 'gait'/exp OR 'gait' OR 'mobility'/exp OR 'mobility' OR 'balance'/exp OR 'balance' OR 'postural balance'/exp OR 'postural balance' OR 'stability'/exp OR 'stability' OR 'fall'/exp OR 'fall' OR 'posture'/exp OR 'posture'
2. 'aged'/exp OR 'frail elderly'/exp OR 'aging' OR 'elders' OR 'geriatric' OR 'older adults' OR 'elderly'
3. 'cognitive training'/exp OR 'cognitive training' OR 'videogame'/exp OR 'videogame' OR 'cognitive stimulation' OR 'cognitive rehabilitation' OR 'cognitive remediation' OR 'memory training' OR 'computer game' OR 'attention training'
4. 1 AND 2 AND 3

Filters:

Language: English

Publication type: Randomized controlled trial

Age groups: [aged]/lim OR [very elderly]/lim)

exp: Medical Subject Headings

**Databases: PsycINFO**

1. (MAINSUBJECT.EXACT("Brai n Training") OR MAINSUBJECT.EXACT("Com puter Games")) OR "cognitive stimulation" OR "cognitive rehabilitation" OR "cognitive remediation" OR "memory training" OR "video games" OR "attention training" OR "cognitive training" OR "computer games"
2. MAINSUBJECT.EXACT("Geria tric Patients") OR "older adults" OR "elderly" OR "aging" OR "aged" OR "elders" OR "frail elderly" OR "geriatric"
3. (MAINSUBJECT.EXACT("Post ure") OR MAINSUBJECT.EXACT("Gait" )) OR "mobility" OR "balance" OR "postural balance" OR "stability" OR "fall" OR "gait" OR "posture"
4. 1 AND 2 AND 3

Filters:

Language: English

Publication type: Randomized controlled trial

Age groups: Aged (65 yrs & older)
