# Supplementary material for: Effects of Pure Cognitive Training on Gait and Balance in Older Adults: Systematic Review and Meta-Analysis
Source: JMIR Aging. 2026 Jul 29;9:e85070. doi: 10.2196/85070 (PMC13469839; doi:10.2196/85070)
Supplement: Supplementary file 2 [file aging_v9i1e85070_app2.docx]

**Table S2.** Downgrade and upgrade criteria of grade

| Criteria used to downgrade and upgrade rating in the GRADE of Recommendation, Assessment, Development and Evaluation (GRADE) system. See the Methods section for further details. | |
| --- | --- |
| Risk of bias | - For outcomes where meta-analysis was possible, more than half of the participants included in the primary analysis from trials with a PEDro score of < 6: downgrade 1 level - For outcomes with more than half of the participants from trials with a PEDro score < 4: downgrade 2 level |
| Inconsistency | - For outcomes where meta-analysis was possible, I^2^ > 50% in the meta-analysis: downgraded 1 level |
| Indirectness | - The participants, intervention, comparator intervention, outcome measure or study design did not match between the included studies and the eligibility criteria for this review: downgrade 1 level |
| Imprecision | - The small effect sizes of 0.2 and -0.2 of the standard mean difference represent the appreciable harm and benefit thresholds, respectively.   - Subjects included in the meta-analysis were less than 400, or the 95%CIs spanned the appreciable harm or benefit thresholds: downgrade 1 level   - Subjects included in the meta-analysis were less than 400, and the 95%CIs spanned both appreciable harm and benefit thresholds: downgrade 2 level |
| Publication bias | - *p*<0.1 on the two-tailed Egger’s regression asymmetry test: downgrade 1 level |
| Large effect | - SMD ≥ 0.8 in the meta-analysis: upgrade 1 level |
| Plausible confounding | - Study design results in the presence of residual confounding that reduced the demonstrated effect: upgrade 1 level |
| Dose response gradient | - Response gradient was detected in meta-regression analysis: upgrade 1 level. |

PEDro score, methodology quality from Physiotherapy Evidence Database;

I^2^, I square value in the meta-analysis;

CIs, Confidence intervals value.
