# Supplementary material for: Effects of Pure Cognitive Training on Gait and Balance in Older Adults: Systematic Review and Meta-Analysis
Source: JMIR Aging. 2026 Jul 29;9:e85070. doi: 10.2196/85070 (PMC13469839; doi:10.2196/85070)
Supplement: Supplementary file 3 [file aging_v9i1e85070_app3.docx]

**Table S3.** Summary of subject characteristics and intervention protocols

| Study | Subject characteristics | | | | | | Intervention group | | | | | | Control group |
| --- | --- | --- | --- | --- | --- | --- | --- | --- | --- | --- | --- | --- | --- |
|  | Inclusion/exclusion criteria | Sample size | Age | Gender | MMSE | With or without balance impairment | Type | Supervision | Inte-nsity | Duration of program | Duration per session | Frequency per session |  |
| **Althomali et al., 2019 [1]** | Inclusion Criteria:   - healthy community-dwelling adults - 70 years or older - Either gender - Visual acuity of 6/12 or better in both eyes   Exclusion Criteria   - Unable to speak English - Cognitive impairment or cognitive delay - Unable to walk independently without walker or cane | n=30;  IG, n=15  CG, n=15 | Total: 80.3 (6)  IG: 78.7 (5.8)  CG: 81.7 (6.1) | M=16  F=14 | NR | Mixed participants. Base line gait speed was not reported  Gait speed tested by 5MWT | Visual attention training in attended field of view (AFOV) and useful field of view test 2 (UFV2) with different difficulties and targets | NR | NR | 3 weeks | 45 mins | 2x/week | Continue their everyday activities as usual |
| **Azadian et al., 2016 [2]** | Inclusion Criteria:   - Male senior adult - BBS<52 - and/or completed a 10m walk with a self-selected gait speed of 1.1m/s or less - Normal or corrected-to-normal vision   Exclusion Criteria:   - MMSE<24 - Any neurological or vestibular disorder or orthopaedic condition including lower limb injury 6 months before data collection | n=20  IG, n=10  CG, n=10 | IG:  73.8 (3.9)  CG:  73.7 (4.4) | M=30  F=0 | IG:  24.1 (1.9)  CG:  24.9 (2.1) | With balance impairment  All subjects were scored less than 52 on the Berg Balance Score (BBS), and/or completed a 10-m walk with a self-selected gait speed of 1.1 m/s or less.  Gait speed test by 6min walk | Mindfit: Computerized training on various spatial and  verbal working memory tasks, i.e., visual, auditory and stabilized tasks each of which contained three parts related to numbers, letters, and shapes. | NR | NR | 8 weeks | 45 mins | 3x/weeks | NR |
| **Blackwood et al., 2016 [3]** | Inclusion Criteria:   - Aged 65 or older - Independent with transfers and ambulation - Able to communicate in English - Independently consent to participate - Live independently in the community - Good vision/ without prescriptive lenses - Positive of computer use history (4 or more hours/week)   Exclusion Criteria:   - cognitive impairment - Receiving healthcare service for mobility impairment - History of (1) cerebrovascular accident; (2) head trauma; (3) TBI; (4) surgery on their leg 6 months prior to testing - Unable to meet the requirement of the test or commit the time of the test   Has received any cognitive training program 9 months prior to testing | n=44  IG, n=19  CG, n=25 | Total: 74.6  IG: 73.79 (4.36)  CG: 75.68 (6.88) | IG: M=14  F=5  CG:  M=18  F=7 | NR | NR balance ability  Baseline self-selected gait speed : 1.06 m/s | Lumosity: Home-based  computerized cognitive training which focused on set shifting, attention, and visual spatial ability | Researcher | NR | 6 weeks | 20-25 mins | 3x/weeks | Control group only took part in measurement |
| **Hou et al, 2022 [4]** | Inclusion criteria:   - aged 60 years or more - 6 years or more of education - MMSE scores of 24 points or higher - no video game or exergame experience in the past 6 months - no regular exercise in the past 6 months   Exclusion Criteria:   - hearing disorder - visual impairments   diagnosed with neurological and/or psychiatric disorders | n=43  IG, n= 22  CG, n=21 | Total:  67.7  IG:67.8  (4.96)  CG:67.5  (4.94) | M=8  F=35 | 28.25 | NR balance ability  Base line gait speed 1.5 m/s(6MWT) | Computerized Cognitive training using a game called Crazy Taxi | NR | NR | 12 weeks | 30 mins | 3x/week | Maintain original lifestyle |
| **Li et al., 2010 [5]** | Inclusion Criteria:   - Aged 70+ - Proficiency in English - Normal or corrected-to-normal vision and hearing   Exclusion Criteria:   - Inability to ambulate without assistive devices - History of neurological or musculoskeletal impairment - Balance problems - Unstable or progressing medical conditions - Medical affecting balance or cognitive abilities | n=21  IG, n=11  CG, n=10 | IG:  74.6 (5.7)  CG:  77.7  (7.1) | IG:  M: 3  F: 8  CG:  M: 4  F: 6 | MoCA:  IG:  27.3  (1.9)  CG:  26.5  (1.4) | Without balance impairment  Base line gait speed tested by 6MWT | Cognitive dual-task training while seated in front of a computer monitor | NR | NR | 5 weeks | NR | At least 2 days apart for each session for total of 9 sessions | No treatment for the same duration as training group |
| **Marusic et al., 2022 [6]** | Inclusion Criteria:   - active and healthy older adults (defined according to self-reported questionnaires) - normal or corrected normal vision - no self-reported history of cardiovascular, neurological, or psychiatric conditions   Exclusion Criteria:   - Symptoms of cognitive decline or MoCA score < 2 - history of falls in the past 12 months - regular heavy alcohol consumption - acute or chronic skeletal, neuromuscular, metabolic and/or cardiovascular disease conditions that may limit mobility | n=63  IG, n=32;  CG, n=31 | Total:  67.7  IG:67.7  (5.8)  CG:67.2  (6.0) | Total:  M=15  F=48  IG:  M=7  F=25  CG:  M=8  F=23 | MoCA  Total:  27.2 (1.7)  IG: 27.4 (1.7)  CG:27.0 (1.7) | Without balance impairment  Baseline gait speed 1.14m/s(self-select) | CogniFit Computerized Cognitive training | Minimum of 2 research assistants | NR | 8 weeks | 40 mins | 3x/weeks | Waitlist control and education |
| **Ng et al., 2015 [7]** | Inclusion criteria:   - Prefrail or frail older aged 65 years and above - Able to ambulate without personal assistance - Living at home.   Exclusion criteria:   - Significant cognitive impairment (MMSE below 23) - Major depression - Severe audiovisual impairment - Progressive, degenerative neurologic disease - Terminal illness with life expectancy <12 months - Participating in other interventional studies - Unavailable to participate for the full duration of the study | n=95  IG, n=50  CG, n=50 | Total:  69.9  IG:69.7  (4.31)  CG:70.1  (5.02) | Total M=34  F=66  IG:  M=12  F=38  CG:  M=22  F=28 | IG:  29.1 (1.29)  CG:  28.6 (1.79) | Without balance impairment  Only report fast gait speed | Cognitive training stimulating short-term memory, and enhancing attention and information-processing skills, and reasoning and problem-solving abilities | NR | NR | 24 weeks  (Booster sessions for the latter 12 weeks) | 120 mins | 1x/weeks | Usual care |
| **Smith-Ray et al.,2015 [8]** | Inclusion criteria:   - Aged 70 years or older - At least one self-reported fall within the last 2 years or a self-reported balance impairment, - Score greater than or equal to 26 on MMSE   Exclusion criteria:   - Presence of severe walking or balance impairment - Previously completed a cognitive training program within the last year - Colorblindness - Plans to begin a balance program during the study period - Self-reported presence of vertigo - Visual acuity of less than 20/80 - Currently using psychotropic medications | n=51  IG, n=27  C, n=24 | Total:  82  IG:82.7  (5.99)  CG:81.1  (6.77) | M=12  F=39 | Total:  28.4 (2.00)  IG: 27.79 (2.54)  CG:  28.75 (1.11) | Without sevre balance impairment  Baseline gait speed 1.11m/s | Computerized cognitive training on executive functions including visuospatial working memory, speed of processing, and inhibition (Insight computer software by Posit Science) | NR | NR | 10 weeks | 60 mins | 3x/weeks | Health education |
| **Smith-Ray, R. L., Makowski-Woidan, B., & Hughes, S. L.** **,2014 [9]** | Inclusion Criteria:   - At least one self-reported fall within 2 years/unable to stand on one leg for >3 sec - 65 or older - MMSE greater or equal to 26   Exclusion Criteria:   - Presence of significant balance/walking impairment - Currently/ recently involved in cognitive training program - Plans to begin a balance program during study period - Currently on psychotropic medication | n=45with history of falls  IG=23  CG=22 | 72.5 | M=4  F=41 | Equal or greater than 26 | Without balance impairment  Baseline gait speed:0.992m/s | Computerized cognitive training on executive functions including visuospatial working memory, speed of processing, and inhibition (Insight computer software by Posit Science) | Research assistant | NR | 10 weeks | 1 hour | 2x/week | No intervention |
| **Verghese et al., 2010 [10]** | Inclusion Criteria:   - 70 years old or older - Ability to make time commitment - Sedentary (physically inactive or exercises once weekly or less) - Gait velocity <1.0 m/s   Exclusion Criteria:   - Presence of dementia (Telephone Memory Impairment Screen score ≤5, MMSE score ≤25, or diagnosed by study clinicians - Inability to ambulate - Hospitalized in past 3 months for severe illness or surgery - Major neurological or psychiatric illness history - Concurrent enrollment in other studies | n=24  IG, n=12  CG, n=12 | IG:  77.4 (7.0)  CG:  79.9  (7.5) | IG:  M=4  F=8  CG:  M=5  F=7 | IG:  29.0  (0.3)  CG:  29.1  (0.4) | Baseline: 0.692m/s(all subjects gait speed <1m/s) | Mindfit: 8-week computerized cognitive remediation program | NR | NR | 8 weeks | 45-60 min | 3x/week, with at least a 1-day interval  between training days | Health education |
| **Steinmetz and Federspiel, 2014 [11]** | Inclusion Criteria:   - Ability to walk unaided for > 10m - No substantial visual or hearing impairment unless corrected - MMSE equal to or greater than 23   Inclusion Criteria:   - Not meeting inclusion criteria | n=21 with  IG= 12  CG= 9 | IG:  83.8 (6.5)  CG:  85.7  (5.4) | IG:  M=2  F=10  CG:  M=2  F=7 | IG:  27.1 ± 1.8  CG:  27.6 ± 2.0 | Baseline gait speed: 0.661m/s | NEUROvitalis: Cognitive training composed of basic theory learning | Experienced trainer | NR | 6 weeks | 90 mins | 2x/weeks | Maintain original lifestyle |
| **Liu Y et al., 2023 [12]** | Inclusion Criteria:   - over 55 years of age; - ambulatory without any assistive devices; - normal or corrected-to-normal vision; - willing to sign the informed consent and to make time commitment   Exclusion Criteria:   - receiving physical therapy; - suffering from cardiovascular and cerebrovascular diseases; - history of head or brain trauma; - having cognitive or potential neurological deficits detected by Mini-Mental State Examination (MMSE). | n=139 with IG=64  CG=75 | 66.2  (7.1) | IG:  M=16  F=48  CG:  M=19  F=56 | NR | Without balance impairment  No gait speed outcome | Flanker test | Researchers | NR | 22 weeks | 30 mins | 3times a week | maintained daily life and regularly and health education |
| **Sturnieks et al., 2024 [13]** | Inclusion criteria:   - age 65 years or older; - English-speaking; - living in the Sydney metropolitan area; - independent in activities of daily - living; - able to walk 10 m without the use of a walking aid; - and willing - to provide informed consent | n=238  IG=118  CG=120 | IG=72.6(5.5)  CG=72.5(5.5) | IG: F=189(72.1%)  CG:  F=182(71.4%) | NR | Without balance impairment  Baseline Gait speed:1.21m/s | custom-built desktop touchpad and trained by playing the  games by pressing target panels with their hands | Researchers | NR | 12months | NR (self-decided by participant) | Weekly from 120 to 150mins | NR |
| **Ammar et al., 2024 [14]** | Inclusion criteria:   - age 65 years or older; - MMSE score of 26 or above - a minimum of one self-reported fall in the previous two years or a Berg balance scale (BBS) score between more than 41 and <52 - greed to devote the necessary amount of time as desired by the program | n=64  IG=32  CG=32 | IG=67.91(3.14)  CG=69.93(4.77) | IG: F=16, M=16  CG: F=17, M=15 | IG: 26.87(0.83)  CG: 27.41(0.87) | Gait speed: NR  BBS score between more than 41 and <52 | Board game cognitive training | Researchers | NR | 8 weeks | 60 min/day | 3 times per week | No intervention applied. |

IG=Intervention Group; CG=control group; MMSE=Mini-Mental State Examination; NR=Not report; CPS= cognitive performance scale; MoCA: The Montreal Cognitive Assessment; TBI: Traumatic Brain Injury; SPPB: Short Physical Performance Battery, DVT: Deep vein thrombosis

References:

1.	Althomali MM, Vallis LA, Leat SJ. Can Older Adults' Balance and Mobility Improve with Visual Attention Training? Eur J Appl Physiol. 2019;119(7):1649-61. PMID: 31055677. doi: 10.1007/s00421-019-04153-2.

2.	Azadian E, Torbati HR, Kakhki AR, Farahpour N. The Effect of Dual Task and Executive Training on Pattern of Gait in Older Adults with Balance Impairment: A Randomized Controlled Trial. Arch Gerontol Geriatr. 2016;62:83-9. PMID: 26508098. doi: 10.1016/j.archger.2015.10.001.

3.	Blackwood J, Shubert T, Fogarty K, Chase C. The Impact of a Home-Based Computerized Cognitive Training Intervention on Fall Risk Measure Performance in Community Dwelling Older Adults, a Pilot Study. J Nutr Health Aging. 2016;20(2):138-45. PMID: 26812509. doi: 10.1007/s12603-015-0598-5.

4.	Hou HY, Li HJ. Effects of Exergame and Video Game Training on Cognitive and Physical Function in Older Adults: A Randomized Controlled Trial. Appl Ergon. 2022;101:103690. PMID: 35066398. doi: 10.1016/j.apergo.2022.103690.

5.	Li KZ, Roudaia E, Lussier M, Bherer L, Leroux A, McKinley PA. Benefits of Cognitive Dual-Task Training on Balance Performance in Healthy Older Adults. J Gerontol A Biol Sci Med Sci. 2010;65(12):1344-52. PMID: 20837662. doi: 10.1093/gerona/glq151.

6.	Marusic U, Verghese J, Mahoney JR. Does Cognitive Training Improve Mobility, Enhance Cognition, and Promote Neural Activation? Front Aging Neurosci. 2022;14:845825. PMID: 35677205. doi: 10.3389/fnagi.2022.845825.

7.	Ng TP, Feng L, Nyunt MS, Feng L, Niti M, Tan BY, et al. Nutritional, Physical, Cognitive, and Combination Interventions and Frailty Reversal Among Older Adults: A Randomized Controlled Trial. Am J Med. 2015;128(11):1225-36. PMID: 26159634. doi: 10.1016/j.amjmed.2015.06.017.

8.	Smith-Ray RL, Hughes SL, Prohaska TR, Little DM, Jurivich DA, Hedeker D. Impact of Cognitive Training on Balance and Gait in Older Adults. J Gerontol B Psychol Sci Soc Sci. 2015;70(3):357-66. PMID: 24192586. doi: 10.1093/geronb/gbt097.

9.	Smith-Ray RL, Makowski-Woidan B, Hughes SL. A Randomized Trial to Measure the Impact of A Community-Based Cognitive Training Intervention on Balance and Gait in Cognitively Intact Black Older Adults. Health Educ Behav. 2014;41(1 Suppl):62s-9s. PMID: 25274713. doi: 10.1177/1090198114537068.

10.	Verghese J, Mahoney J, Ambrose AF, Wang C, Holtzer R. Effect of Cognitive Remediation on Gait in Sedentary Seniors. J Gerontol A Biol Sci Med Sci. 2010;65(12):1338-43. PMID: 20643703. doi: 10.1093/gerona/glq127.

11.	Steinmetz JP, Federspiel C. The Effects of Cognitive Training on Gait Speed and Stride Variability in Old Adults: Findings from a Pilot Study. Aging Clin Exp Res. 2014;26(6):635-43. PMID: 24803285. doi: 10.1007/s40520-014-0228-9.

12.	Liu Y, Zheng M, Liu J, Cao C, Ding Y. Effects of Cognitive Training in Flanker Task on Mobility of Older Adults: A Randomized Controlled Trail. Gerontology. 2023;69(5):593-602. PMID: 36646052. doi: 10.1159/000529088.

13.	Sturnieks DL, Hicks C, Smith N, Ratanapongleka M, Menant J, Turner J, et al. Exergame and Cognitive Training for Preventing Falls in Community-Dwelling Older People: A Randomized Controlled Trial. Nat Med. 2024;30(1):98-105. PMID: 38228913. doi: 10.1038/s41591-023-02739-0.

14.	Ammar M, Amjad I, Nisar M, Ghoneim OSA. Effects of Board Games on Balance in Association with Cognition in Community-Dwelling Older Adults. J Musculoskelet Surg Res. 2024;8(3):256-63. doi: 10.25259/JMSR_122_2024.
