# Supplementary material for: Effects of Pure Cognitive Training on Gait and Balance in Older Adults: Systematic Review and Meta-Analysis
Source: JMIR Aging. 2026 Jul 29;9:e85070. doi: 10.2196/85070 (PMC13469839; doi:10.2196/85070)
Supplement: Supplementary file 4 [file aging_v9i1e85070_app4.docx]

**Table S4.** Summary of intervention effects

| **Study** | **Results** | |
| --- | --- | --- |
|  | **Intra-group** | **Inter-group** |
| **Althomali et al., 2019 [1]** | OLST  IG: - (p=0.33)    Mini-BESTest  IG: - (p=0.078)    TUG  IG: - (p=0.07)    TUG with cognitive dual task  IG: - (p=0.66)    Gait speed (5MWT)  IG: - (p=0.48) | OLST  IG~CG (p=0.90)    Mini-BESTest  IG~CG (p=0.58)    TUG  IG~CG (p=0.3)    TUG with cognitive dual task  IG~CG (p=0.53)    Gait speed (5MWT)  IG~CG (p=0.38) |
| **Azadian et al., 2016 [2]** | Gait speed  IG: ↑ (p<0.001)    Cadence  IG: ↑ (p<0.001)    Stride time  IG: ↑ (p=0.00)    Single support time  IG: ↑ (p=0.00)    Swing time  IG: ↑ (p=0.00)    Double support time  IG: ↑ (p=0.00)    Stride length  IG: - (p>0.05) | Gait speed  IG > CG (p=0.001)    Cadence  IG > CG (p<0.001)    Stride time  IG > CG (p<0.00)    Single support time  IG > CG (p<0.001)    Swing time  IG > CG (p=0.00)    Double support time  IG > CG (p<0.016)    Stride length  IG ~ CG (p>0.05) |
| **Blackwood et al., 2016 [3]** | TUG :  NR    Gait speed:  NR | TUG  CG ~ IG (p=0.38)    Gait speed  IG > CG (p=0.03) |
| **Hou et al., 2022 [4]** | Balance (TUG + OLST with eye-closed)  IG: - (p=0.13) | Balance  IG ~ CG (p=0.56) |
| **Li et al., 2010** **[5]** | Gait speed  NR    Dual-task gait speed (0-back)  NR    Dual-task gait speed (2-back)  NR | Gait speed  IG ~ CG (p=0.103)    Dual-task gait speed (0-back)  NR    Dual-task gait speed (2-back)  NR |
| **Marusic et al., 2022 [6]** | Self-Selected Single-Task Gait Speed  IG: ↑ (p<0.001)    Self-Selected Dual-Task Gait Speed  IG: ↑ (p<0.001) | Self-Selected Single-task Gait Speed  IG ~ CG (p=0.057)    Self-Selected Dual task Gait Speed  IG > CG (p=0.008) |
| **Ng et al, 2015 [7]** | Fast Gait speed  IG: - (p>0.05) | Fast Gait speed  CG ~ IG (p>0.05) |
| **Smith-Ray et al., 2015 [8]** | TUG  IG: -    Gait speed  IG: -    Dual-task gait speed  IG: - | TUG  IG > CG (p=0.036)    Gait speed  IG ~ CG (p=0.124)    Dual-task gait speed  IG ~ CG (p=0.195) |
| **Smith-Ray, R. L., Makowski-Woidan, B., & Hughes, S. L. ,2014 [9]** | BBS  IG: -    Gait speed  IG: -    Dual-task gait speed  IG: - | BBS  IG > CG (p=0.038)    Gait speed  IG > CG (p=0.016)    Dual-task gait speed  IG ~ CG (p=0.659) |
| **Verghese et al., 2010 [10]** | Gait speed  IG: ↑ (p = 0.05)      Gait speed while talking  IG: ↑ (p = 0.002) | Gait speed  IG ~ CG (p=0.10)      Gait speed while talking  IG ~ CG (p=0.05) |
| **Steinmetz and Federspiel**  **2014 [11]** | Single walking gait speed  IG: -    Simple dual-task gait speed  IG: -    Complex dual-task gait speed  IG: - | Single walking gait speed  IG ~ CG    Simple dual-task gait speed  IG ~ CG    Complex dual-task gait speed  IG ~ CG |
| **Liu Y et.al**  **2023 [12]** | Balance (Single leg balance with eyes closed):  IG: (p < 0.001)  Single walking gait speed(TUG)  IG: (p < 0.001) | Balance (Single leg balance with eyes closed):  IG ~ CG (p< 0.01)  Single walking gait speed(TUG)  IG ~ CG (p< 0.001) |
| **Sturnieks et al., 2024 [13]** | Gait speed  IG: NR    Dual-task gait speed  IG: NR | Gait speed  IG ~ CG Not significant  Dual-task gait speed  IG ~ CG Not significant |
| **Ammar et al., 2024 [14]** | BBS  IG: ↑(p=0.014)  TUG  IG: ↑(p < 0.001) | BBS  IG ~ CG (p=0.09)  TUG  IG ~ CG (p=0.834) |

“↑” indicates improvement, not necessarily increase in score. “-” indicates no significant change. “>” indicates “better” in between group comparisons. “~” indicates no significant difference in between group comparisons. NR= Not Reported, IG=Intervention Group; CG=Control Group, TUG: Timed Up & Go, OLST: One-Legged Stance Test, 5MWT: Five-Minute Walk Test, BBS: Berg Balance Scale

References:

1.	Althomali MM, Vallis LA, Leat SJ. Can Older Adults' Balance and Mobility Improve with Visual Attention Training? Eur J Appl Physiol. 2019;119(7):1649-61. PMID: 31055677. doi: 10.1007/s00421-019-04153-2.

2.	Azadian E, Torbati HR, Kakhki AR, Farahpour N. The Effect of Dual Task and Executive Training on Pattern of Gait in Older Adults with Balance Impairment: A Randomized Controlled Trial. Arch Gerontol Geriatr. 2016;62:83-9. PMID: 26508098. doi: 10.1016/j.archger.2015.10.001.

3.	Blackwood J, Shubert T, Fogarty K, Chase C. The Impact of a Home-Based Computerized Cognitive Training Intervention on Fall Risk Measure Performance in Community Dwelling Older Adults, a Pilot Study. J Nutr Health Aging. 2016;20(2):138-45. PMID: 26812509. doi: 10.1007/s12603-015-0598-5.

4.	Hou HY, Li HJ. Effects of Exergame and Video Game Training on Cognitive and Physical Function in Older Adults: A Randomized Controlled Trial. Appl Ergon. 2022;101:103690. PMID: 35066398. doi: 10.1016/j.apergo.2022.103690.

5.	Li KZ, Roudaia E, Lussier M, Bherer L, Leroux A, McKinley PA. Benefits of Cognitive Dual-Task Training on Balance Performance in Healthy Older Adults. J Gerontol A Biol Sci Med Sci. 2010;65(12):1344-52. PMID: 20837662. doi: 10.1093/gerona/glq151.

6.	Marusic U, Verghese J, Mahoney JR. Does Cognitive Training Improve Mobility, Enhance Cognition, and Promote Neural Activation? Front Aging Neurosci. 2022;14:845825. PMID: 35677205. doi: 10.3389/fnagi.2022.845825.

7.	Ng TP, Feng L, Nyunt MS, Feng L, Niti M, Tan BY, et al. Nutritional, Physical, Cognitive, and Combination Interventions and Frailty Reversal Among Older Adults: A Randomized Controlled Trial. Am J Med. 2015;128(11):1225-36. PMID: 26159634. doi: 10.1016/j.amjmed.2015.06.017.

8.	Smith-Ray RL, Hughes SL, Prohaska TR, Little DM, Jurivich DA, Hedeker D. Impact of Cognitive Training on Balance and Gait in Older Adults. J Gerontol B Psychol Sci Soc Sci. 2015;70(3):357-66. PMID: 24192586. doi: 10.1093/geronb/gbt097.

9.	Smith-Ray RL, Makowski-Woidan B, Hughes SL. A Randomized Trial to Measure the Impact of A Community-Based Cognitive Training Intervention on Balance and Gait in Cognitively Intact Black Older Adults. Health Educ Behav. 2014;41(1 Suppl):62s-9s. PMID: 25274713. doi: 10.1177/1090198114537068.

10.	Verghese J, Mahoney J, Ambrose AF, Wang C, Holtzer R. Effect of Cognitive Remediation on Gait in Sedentary Seniors. J Gerontol A Biol Sci Med Sci. 2010;65(12):1338-43. PMID: 20643703. doi: 10.1093/gerona/glq127.

11.	Steinmetz JP, Federspiel C. The Effects of Cognitive Training on Gait Speed and Stride Variability in Old Adults: Findings from a Pilot Study. Aging Clin Exp Res. 2014;26(6):635-43. PMID: 24803285. doi: 10.1007/s40520-014-0228-9.

12.	Liu Y, Zheng M, Liu J, Cao C, Ding Y. Effects of Cognitive Training in Flanker Task on Mobility of Older Adults: A Randomized Controlled Trail. Gerontology. 2023;69(5):593-602. PMID: 36646052. doi: 10.1159/000529088.

13.	Sturnieks DL, Hicks C, Smith N, Ratanapongleka M, Menant J, Turner J, et al. Exergame and Cognitive Training for Preventing Falls in Community-Dwelling Older People: A Randomized Controlled Trial. Nat Med. 2024;30(1):98-105. PMID: 38228913. doi: 10.1038/s41591-023-02739-0.

14.	Ammar M, Amjad I, Nisar M, Ghoneim OSA. Effects of Board Games on Balance in Association with Cognition in Community-Dwelling Older Adults. J Musculoskelet Surg Res. 2024;8(3):256-63. doi: 10.25259/JMSR_122_2024.
