# Supplementary material for: Effects of Pure Cognitive Training on Gait and Balance in Older Adults: Systematic Review and Meta-Analysis
Source: JMIR Aging. 2026 Jul 29;9:e85070. doi: 10.2196/85070 (PMC13469839; doi:10.2196/85070)
Supplement: Supplementary file 5 [file aging_v9i1e85070_app5.docx]

**Table S5.** PEDro score of included articles

| Criteria | Althomali et al [1] | Azadian et al [2] | Ammar et al [3] | Blackwood et al [4] | Hou & Li [5] | Li et al [6] | Marusic et al [7] | Ng et al [8] | Smith-Ray et al [9] | Smith-Ray et al [10] | Verghese et al [11] | Steinmetz & Federspiel [12] | Liu et al [13] | Sturnieks et al [14] |
| --- | --- | --- | --- | --- | --- | --- | --- | --- | --- | --- | --- | --- | --- | --- |
| Eligibility criteria | 1 | 1 | 1 | 1 | 1 | 1 | 1 | 1 | 1 | 1 | 1 | 1 | 1 | 1 |
| Random allocation | 1 | 1 | 1 | 0 | 1 | 1 | 1 | 1 | 1 | 1 | 1 | 1 | 1 | 1 |
| Concealed allocation | 0 | 0 | 0 | 0 | 0 | 1 | 0 | 1 | 0 | 0 | 0 | 1 | 1 | 1 |
| Baseline comparability | 1 | 1 | 0 | 1 | 1 | 1 | 1 | 1 | 1 | 1 | 1 | 1 | 1 | 1 |
| Blind subjects | 0 | 0 | 0 | 0 | 0 | 0 | 1 | 0 | 0 | 0 | 1 | 0 | 0 | 0 |
| Blind therapists | 1 | 0 | 0 | 0 | 0 | 0 | 0 | 0 | 0 | 0 | 0 | 0 | 0 | 0 |
| Blind assessors | 1 | 0 | 0 | 0 | 1 | 0 | 1 | 1 | 0 | 1 | 1 | 0 | 0 | 1 |
| Adequate follow-up | 1 | 1 | 1 | 1 | 0 | 1 | 1 | 1 | 1 | 0 | 0 | 0 | 0 | 1 |
| Intention-to-treat analysis | 1 | 0 | 0 | 0 | 0 | 1 | 1 | 1 | 1 | 0 | 0 | 0 | 1 | 1 |
| Between-group comparisons | 1 | 1 | 1 | 1 | 1 | 1 | 1 | 1 | 1 | 1 | 1 | 1 | 1 | 1 |
| Point estimates and variability | 1 | 1 | 1 | 1 | 1 | 1 | 1 | 1 | 1 | 1 | 1 | 1 | 1 | 1 |
| **Total** | **8** | **5** | **4** | **4** | **5** | **7** | **8** | **8** | **6** | **5** | **6** | **5** | **6** | **8** |

A score of 1, the study fulfilled the criterion; A score of 0, the study did not fit the criterion.

References:

1.	Althomali MM, Vallis LA, Leat SJ. Can Older Adults' Balance and Mobility Improve with Visual Attention Training? Eur J Appl Physiol. 2019;119(7):1649-61. PMID: 31055677. doi: 10.1007/s00421-019-04153-2.

2.	Azadian E, Torbati HR, Kakhki AR, Farahpour N. The Effect of Dual Task and Executive Training on Pattern of Gait in Older Adults with Balance Impairment: A Randomized Controlled Trial. Arch Gerontol Geriatr. 2016;62:83-9. PMID: 26508098. doi: 10.1016/j.archger.2015.10.001.

3.	Ammar M, Amjad I, Nisar M, Ghoneim OSA. Effects of Board Games on Balance in Association with Cognition in Community-Dwelling Older Adults. J Musculoskelet Surg Res. 2024;8(3):256-63. doi: 10.25259/JMSR_122_2024.

4.	Blackwood J, Shubert T, Fogarty K, Chase C. The Impact of a Home-Based Computerized Cognitive Training Intervention on Fall Risk Measure Performance in Community Dwelling Older Adults, a Pilot Study. J Nutr Health Aging. 2016;20(2):138-45. PMID: 26812509. doi: 10.1007/s12603-015-0598-5.

5.	Hou HY, Li HJ. Effects of Exergame and Video Game Training on Cognitive and Physical Function in Older Adults: A Randomized Controlled Trial. Appl Ergon. 2022;101:103690. PMID: 35066398. doi: 10.1016/j.apergo.2022.103690.

6.	Li KZ, Roudaia E, Lussier M, Bherer L, Leroux A, McKinley PA. Benefits of Cognitive Dual-Task Training on Balance Performance in Healthy Older Adults. J Gerontol A Biol Sci Med Sci. 2010;65(12):1344-52. PMID: 20837662. doi: 10.1093/gerona/glq151.

7.	Marusic U, Verghese J, Mahoney JR. Does Cognitive Training Improve Mobility, Enhance Cognition, and Promote Neural Activation? Front Aging Neurosci. 2022;14:845825. PMID: 35677205. doi: 10.3389/fnagi.2022.845825.

8.	Ng TP, Feng L, Nyunt MS, Feng L, Niti M, Tan BY, et al. Nutritional, Physical, Cognitive, and Combination Interventions and Frailty Reversal Among Older Adults: A Randomized Controlled Trial. Am J Med. 2015;128(11):1225-36. PMID: 26159634. doi: 10.1016/j.amjmed.2015.06.017.

9.	Smith-Ray RL, Hughes SL, Prohaska TR, Little DM, Jurivich DA, Hedeker D. Impact of Cognitive Training on Balance and Gait in Older Adults. J Gerontol B Psychol Sci Soc Sci. 2015;70(3):357-66. PMID: 24192586. doi: 10.1093/geronb/gbt097.

10.	Smith-Ray RL, Makowski-Woidan B, Hughes SL. A Randomized Trial to Measure the Impact of A Community-Based Cognitive Training Intervention on Balance and Gait in Cognitively Intact Black Older Adults. Health Educ Behav. 2014;41(1 Suppl):62s-9s. PMID: 25274713. doi: 10.1177/1090198114537068.

11.	Verghese J, Mahoney J, Ambrose AF, Wang C, Holtzer R. Effect of Cognitive Remediation on Gait in Sedentary Seniors. J Gerontol A Biol Sci Med Sci. 2010;65(12):1338-43. PMID: 20643703. doi: 10.1093/gerona/glq127.

12.	Steinmetz JP, Federspiel C. The Effects of Cognitive Training on Gait Speed and Stride Variability in Old Adults: Findings from a Pilot Study. Aging Clin Exp Res. 2014;26(6):635-43. PMID: 24803285. doi: 10.1007/s40520-014-0228-9.

13.	Liu Y, Zheng M, Liu J, Cao C, Ding Y. Effects of Cognitive Training in Flanker Task on Mobility of Older Adults: A Randomized Controlled Trail. Gerontology. 2023;69(5):593-602. PMID: 36646052. doi: 10.1159/000529088.

14.	Sturnieks DL, Hicks C, Smith N, Ratanapongleka M, Menant J, Turner J, et al. Exergame and Cognitive Training for Preventing Falls in Community-Dwelling Older People: A Randomized Controlled Trial. Nat Med. 2024;30(1):98-105. PMID: 38228913. doi: 10.1038/s41591-023-02739-0.
