# Supplementary material for: Effects of Pure Cognitive Training on Gait and Balance in Older Adults: Systematic Review and Meta-Analysis
Source: JMIR Aging. 2026 Jul 29;9:e85070. doi: 10.2196/85070 (PMC13469839; doi:10.2196/85070)
Supplement: Supplementary file 6 [file aging_v9i1e85070_app7.docx]

Multimedia Appendix 7. Subgroup meta-analyses for functional mobility to seek the source of heterogeneity.

Figure 1. Subgroup analysis based on methodological quality of the effect of pure cognitive training on functional mobility

Figure 2. Subgroup analysis based on the type of training (CCT vs non-CCT) of the effect of pure cognitive training on functional mobility

Figure 3. Subgroup analysis based on total training hours of the effect of pure cognitive training on functional mobility

Figure 4. Subgroup analysis based on individual training duration of the effect of pure cognitive training on functional mobility

Figure 5. Subgroup analysis based on baseline mean age of the effect of pure cognitive training on functional mobility
